# Bilateral Fibular Graft: Biological Reconstruction after Resection of Primary Malignant Bone Tumors of the Lower Limb

**DOI:** 10.1155/2013/205832

**Published:** 2013-04-16

**Authors:** Maya Niethard, Carmen Tiedke, Dimosthenis Andreou, Frank Traub, Mario Kuhnert, Mathias Werner, Per-Ulf Tunn

**Affiliations:** ^1^Centre of Orthopedic and Trauma Surgery, Department of Oncologic Surgery, Sarcoma Centre Berlin-Brandenburg, HELIOS Klinikum Berlin Buch, Schwanebecker Chaussee 50, 13125 Berlin, Germany; ^2^Department of Vascular Surgery, Sarcoma Centre Berlin-Brandenburg, HELIOS Klinikum Berlin Buch, Schwanebecker Chaussee 50, 13125 Berlin, Germany; ^3^Department of Pathology, Sarcoma Centre Berlin-Brandenburg, HELIOS Klinikum Emil von Behring, Walterhöferstraße 11, 14165 Berlin, Germany

## Abstract

This paper deals with bilateral vascularized fibular grafts (BVFG) as a method for reconstruction of metadiaphyseal defects of the femur and tibia in young patients suffering from malignant bone tumors of the lower limb. This reconstructional technique was used in 11 patients undergoing metadiaphyseal resection of lower limb malignant bone tumors. All patients with Ewing's sarcoma and osteosarcoma had multimodal treatment according to the EURO-E.W.I.N.G 99 or COSS-96 protocol. Median FU was 63 months. None of the patients experienced local recurrence during FU. 2 patients died due to distant disease during FU. Full weight- bearing was permitted after a mean of 8 months. The median MSTS score was 87%. Complications occurred in five patients. None of the complications led to failure of the biological reconstruction or to amputation. Biological reconstruction of osseous defects is always desirable when possible and aims at a permanent solution. Good functional and durable results can be obtained by using BVFG for the reconstruction of metadiaphyseal defects of the femur and tibia. Radiotherapy in the multimodal setting increases the risk for graft or fixation failure.

## 1. Introduction

The prognosis of patients with primary malignant bone tumors has improved within the last 30 years also due to the framework of standard therapy optimization studies (EURO E.W.I.N.G 99, COSS-96). In parallel, the proportion of limb-sparing resection and reconstruction procedures has increased steadily. More than 80% of patients can receive limb-sparing resection [1–4] without having an increased risk of local recurrence. The spectrum of reconstruction possibilities is extensive with options as tumor arthroplasty [3, 5–7], massive allografts [8–13], mantle grafts (massive allograft combined with a vascularized fibular graft [2, 14, 15] or irradiated autograft combined with a vascularized fibular graft [16–18]), and biological methods such as fibular grafts (vascularized/nonvascularized, unilateral/bilateral, “double barrel fibula”) [4, 19–28], tibial flake, pelvis flake, and callus distraction [12, 29, 30].

About 65% of primary malignant bone tumors are located in the lower extremities and near to a joint. In German speaking countries arthroplasty is the most common treatment in reconstruction of osseous tumor defects. The 10-year survival rate of endoprothesis is 55%–71% [3, 5–7]. Since it is mostly young patients, revision surgery should be expected in most cases of cured patients. Biological reconstruction techniques should be implemented whenever possible. The goal is a lasting reintegration and modeling in the area of the graft recipient while keeping functional integrity of the donor site.

The present study presents indication, methods, functional outcome and problems of bilateral fibular grafts for defect reconstruction after resection of primary malignant bone tumors in the long bones of the lower extremity.

## 2. Materials and Methods

### 2.1. Patients

Between November 2000 and December 2011, 11 consecutive patients needed a resection of a primary malignant bone tumor of the lower extremity (group of Ewing's sarcoma *n* = 7, osteosarcoma *n* = 1, chondrosarcoma *n* = 1, adamantinoma *n* = 2) and received a defect reconstruction using a bilateral fibular graft. Patients with an Ewing's sarcoma and osteosarcoma were treated according to EURO-E.W.I.N.G.-99 or COSS-96 protocol. Patients included 5 females and 6 males with an age range from 4 to 43 years at the time of surgery (mean age 14 years). All patients presented with tumor stage II B (UICC). Tumors were located in the metadiaphysis of the tibia (*n* = 6) and femur (*n* = 5). The length of the reconstructed defect ranged from 8 to 24,5 cm (median 16 cm). One of the patients underwent neoadjuvant radiotherapy and two other patients received adjuvant radiotherapy as part of the EURO-E.W.I.N.G.-99-protocol (Table 1). The median follow-up was 62 months.

### 2.2. Reconstructive Methods

Reconstructive approach varied depending on the location. In tibial defects (*n* = 6) the ipsilateral fibula was swivelled into the defect after resection of malignant bone tumor leaving the original blood supply intact. The vessels of the contralateral fibular graft were microscopically anastomosed end-to-side upon the a. and v. tibialis anterior in the majority of cases. The fixation of the fibular grafts was achieved by standard plating (AO) as exemplary shown in Figure 2. In two cases an additional medial gastrocnemius flap was used to cover the ventral side of the fibular graft.

For reconstruction of femoral defects (*n* = 5) two free fibular grafts of the ipsilateral and contralateral sides were used. Both grafts were positioned into the osseous defect and fixed with a condylar plate (AO) followed by microscopically assisted vascular anastomoses. Branches of the profound femoral artery and vein served as donor vessels. The peroneal artery was anastomosed in Y technique at both grafts. Each fibular vein was anastomosed separately for the grafts. One patient needed a custom made condylar plate (length: 43 cm) due to a defect size of 23 cm that had to be reconstructed. No preoperative angiography was performed in any of the patients with normal clinical vascular status. The only postoperative imaging carried out was X-ray.

After a complete ease of the affected limb for 6 weeks weight bearing was initiated with 15 kg starting at the 7th postoperative week. Weight bearing was increased in intervals correlating to the radiological examination outcome. Within the first postoperative year clinical and radiological follow-up was performed in 2- to 3-month intervals. 

## 3. Results

This is a retrospective analysis, based on the clinic's internal bone tumor registry database and the evaluation of the medical records. Two patients died of their disease. The remaining 9 patients are regularly seen for follow-up. Median values were calculated, and the MSTS score was provided [31].

### 3.1. Oncological Results

In 10 patients, an R0 resection was achieved, and local tumor recurrence did not occur. Resection in patient 11 (adamantinoma) resulted in an R1 resection showing no evidence of disease at follow-up of 12 months.

Patient 6—with Ewing's sarcoma of the proximal femur—developed multiple bone metastasis 18 months after completion of the multimodal treatment. After a second- and later on third-line chemotherapy, the patient died.

Patient 4—with a Ewing's sarcoma of the tibia—showed multiple bone metastases after 22 months an got a second-line chemotherapy. The patient died.

The remaining nine patients showed no evidence of disease at the end of follow-up (Ewing's sarcoma *n* = 5, osteosarcoma *n* = 1, chondrosarcoma *n* = 1, adamantinoma *n* = 2).

Weight bearing was increased depending on the postoperative radiographic findings in all patients. The full load on the affected limb was released after 4–18 months (median 8 months) (Table 2).

The functional outcome was evaluated using the MSTS score [31] ranging from 60 to 100% with a median of 87%.

### 3.2. Complications

Four out of eleven patients needed one or more surgical revisions.

Patient 2 suffered from a postoperative arterial bleeding of the vascular anastomosis, which was revised within 10 hours postoperatively. The later healing was uneventful.

Patient 3 suffered from a condylar plate failure at the transition site of fibular graft and proximal femur with delayed bone union 21 months postoperatively. The patient had received adjuvant radiotherapy (regression grade III according to Salzer-Kuntschik). The proximal plate fragment was removed and a reosteosynthesis with a condylar plate and autologous cancellous bone graft was performed. Five months postoperatively, full weight bearing was released again. Only 6 months later she suffered a second plate failure resulting in a reosteosynthesis with another condylar plate. Another 6 months later she suffered a third plate failure. The patient was put into an orthesis with tubercular contact and the condylar plate was removed and replaced by a custom made osteosynthesis plate accompanied by autologous cancellous bone graft. There have been no more complications for the following 80 months until today's follow-up.

Patient 4 suffered from a fracture of the fibular graft after reconstruction of the proximal tibia, which healed with conservative treatment in cast immobilization. The patient had received neoadjuvant radiotherapy.

Patient 5 suffered from infection of the osteosynthesis site with synchronous nonunion between the bilateral fibular graft and the distal tibia 3 months postoperatively in relation to adjuvant chemotherapy. The plate was removed followed by surgical debridement and immobilisation in a cast. After healing of the infection adjuvant chemotherapy was continued. Six weeks after the completion of adjuvant chemotherapy the nonunion was resected and an autologous bone graft was performed followed by a lateral reosteosynthesis. Full weight bearing was released 12 weeks postoperatively when complete osseous union was documented by plain X-ray.

Patient 8 suffered from a Ewing's sarcoma of the right femur diaphysis. The biological reconstruction was realised with a bilateral free fibular graft and lateral plate fixation for a defect of 16,5 cm (Figure 3(a)). 15 months after tumor resection a plate fracture occurred at the distal interphase between fibular graft and femur metaphysis combined with a nonunion. The patient had received his neoadjuvant and adjuvant chemotherapy according to EURO-E.W.I.N.G.-99-protocol including adjuvant radiotherapy (Figure 3(b)). The fracture was treated with replating and autogenous bone grafting (Figure 3(c)). 5 months later a second plate fracture occurred on a different level. The fibular grafts themselves showed two fractures on different levels. The bony structures showed signs of demineralization and irregularities due to administered chemo- and radiotherapy (Figure 3(d)). Surgical revision resulted in double plating and autogenous bone grafting. So far there have been no more complications until the last follow-up at 62 months (Figure 3(e)).

**Figure 1:**
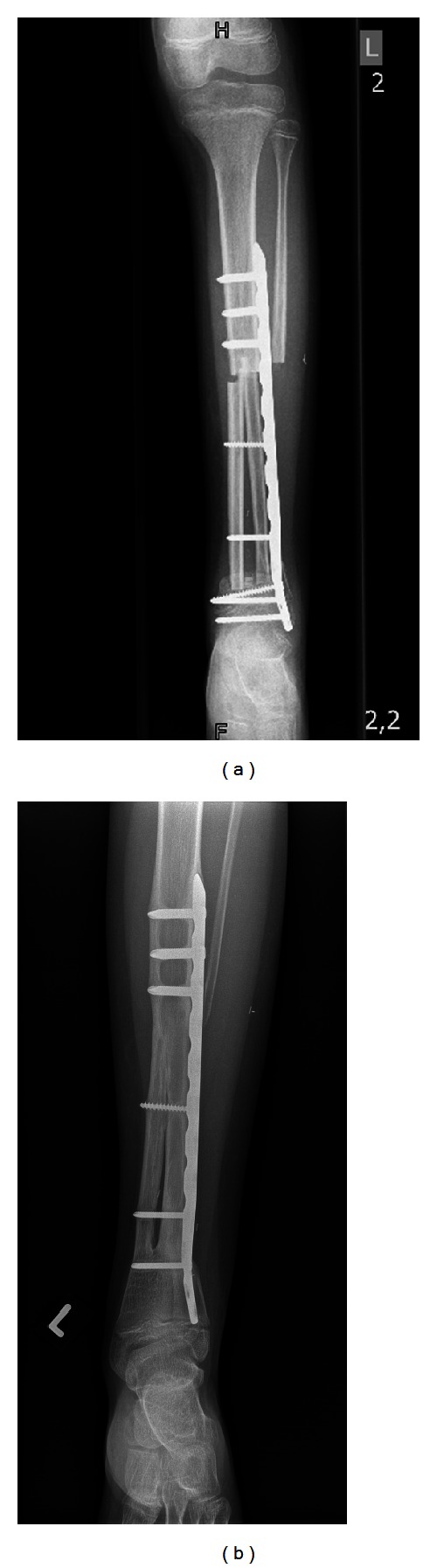
(Patient no. 7) (a) Radiograph of a 9-year-old girl with Ewing's sarcoma of the distal tibia diaphysis. Defect reconstruction (12 cm) was achieved by using bilateral fibular graft and plate osteosynthesis. Due to the small remaining distal epiphyseal fragment the screws had to be placed in the epiphysis. (b) Radiographic results 29 months after tumor resection giving good evidence of bony healing. The epiphyseal screws have been removed. Nevertheless the ankle shows a mild valgus deformity resulting in an MSTS score of 93%.

**Figure 2:**
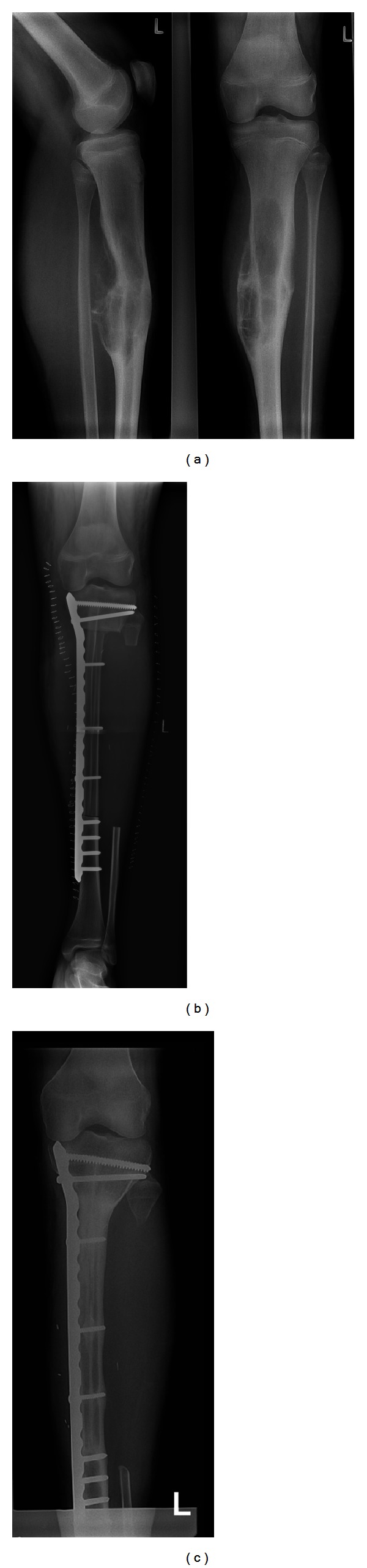
(Patient no. 9) (a) 14-year-old boy with Ewing's sarcoma of the left tibia proximal diaphysis. (b) After completion of neoadjuvant chemotherapy wide resection and reconstruction of the defect (24.5 cm) by a vascularized transposed ipsilateral and contralateral free fibula and medial plate fixation were realised. (c) Radiograph showing osseous integration and hypertrophy of fibular grafts 15 months after operation.

**Figure 3:**
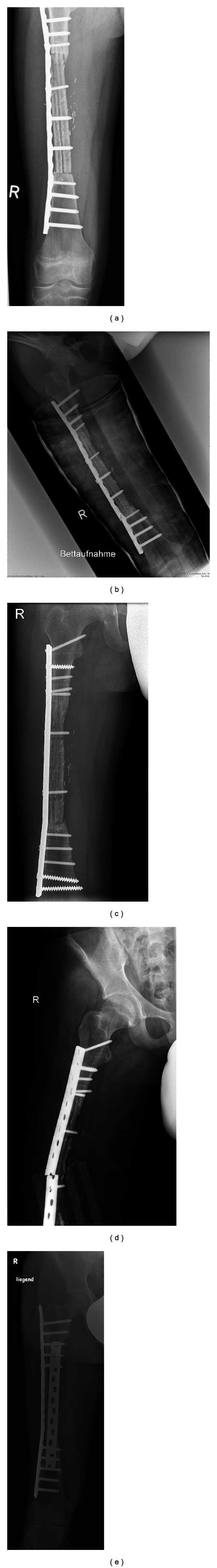
(Patient no. 8) (a) 12-year-old boy with Ewing's sarcoma of the right femur diaphysis. Radiograph showing the reconstructive result with a bilateral free fibular graft and lateral plate fixation for a defect of 16,5 cm. (b) 15 months after tumor resection plate failure occurred at the distal interphase between fibular graft and femur metaphysis showing an osseous nonunion. The patient had received his neoadjuvant and adjuvant chemotherapy according to EURO-E.W.I.N.G.-99-protocol including adjuvant radiotherapy. (c) The fracture was treated with replating and autogenous bone grafting. (d) 5 months later a second plate fracture occurred in the middle of the fibular grafts. The fibular grafts themselves showed two fractures on different levels. The bony structures show signs of demineralization and irregularities due to administered chemo- and radiotherapy. (e) Surgical revision resulting in double plate osteosynthesis and autogenous bone grafting. So far there have been no more complications until the last follow-up at 62 months.

In patient 7 the distal screws for osteosynthesis had to be placed in the epiphysis due to the small remaining distal epiphyseal fragment (Figure 1(a)). The epiphyseal screws were removed 15 months after primary surgery (Figure 1(b)). This was not considered a complication.

There were no signs of donor site morbidity (e.g., Peroneal nerve Paloy, deformity of the tibia, ankle instability). None of the complications resulted in a loss of the affected extremity.

## 4. Discussion

Tumor arthroplasty will remain the most common reconstructive method of near-joint defects caused by resection of primary malignant bone tumors. 5- and 10-year survival rates of arthroplasty are 67%–87% and 55%–71%, respectively [3, 5–7]. Advantages of tumor arthroplasty are the primary stability and good functionality of the limb. Since the majority of the patients are young, revision surgery is undesirable but may be an unavoidable consequence if there is no evidence of disease. In particular infections, aseptic loosening, wear of the joint components and stress fractures are causes for revision surgery. Each intervention increases the risk for a new complication that in the worst case may result in the loss of the affected extremity. 

Massive allografts have similarly high rates of complications including mainly nonunion and infection [8–11]. Mantle grafts (as described in the Capanna's method [33]) represent a fusion between biological and allograft reconstruction. Particularly for the reconstruction of long bone metaphyseal defects an “allograft-mantled” unilateral vascularized fibular graft can enable a high primary stability and in addition promote the bony consolidation of the fibula in the recipient area. Therefore mantle grafts are frequently used in the lower extremity [34–36] but require a bone bank for matching allografts. Postoperative complications include fractures, nonunions, and infections [2, 13–15, 36, 37]. Current available data show that mantle grafts provide a higher primary stability compared to bilateral fibular grafts [14, 15]. 

The advantage of bilateral fibular grafts in long bone metadiaphyseal defect reconstruction of the lower extremity is the autologous transplant which provides excellent chances for remodeling at the recipient's site [21, 23, 25, 28, 38–40]. Particularly reconstruction of femoral defects shows good results with less complications (infections, nonunions) in both procedures compared to the reconstruction of tibial defects. However, the currently available data does not answer the question which reconstruction method (mantle graft versus bilateral fibular graft) provides better results in the long-term survey. 

### 4.1. Stability and Weight Bearing

Biological reconstructions of osseous defects claim to be a permanent solution. Unilateral vascularized and nonvascularized fibulas are used for various reconstructions of the upper and lower extremities. The advantage of vascularized grafts was clearly demonstrated based on animal studies and in clinical trials [4, 27, 39]. From a functional perspective the aim of reconstruction of the lower limb should be to allow early weigth bearing. In the immediate postoperative course a long time of none or partial weight-bearing of the affected limb has to be accepted. This unavoidable restriction can lead to complications such as muscular insufficiency, demineralization of the original bone and graft and pathological fractures [26, 37, 41]. The fibula as an unilateral transplant can only partially provide primary stability in reconstruction of long lower limb bone defects. Weight bearing can sometimes not be permitted for a long time until a hypertrophy of the unilateral fibular graft has taken place [14, 21, 42]. The younger the patient is, the earlier the bone remodelling is expected to take place. Therefore an ipsilateral fibula only as “fibula per tibia” for reconstruction in young patients with malignant tibial bone tumors is adequate since hypertrophy of the fibular graft is expected [21].

Besides the mantle graft which represents a combination of an autologous fibular graft and an allograft the primary stability can be reached and even improved without allogenic transplants by a bilateral fibular graft (fibula per tibia plus free fibular graft of the contralateral side for tibial defects or bilateral free fibular graft for femoral defects). The aim is to reduce time until recovery. This reconstruction method is more complex regarding its surgical technique compared to tumor arthroplasty or unilateral fibular graft and has been repeatedly described in detail [4, 20, 25, 27, 28]. The ipsilateral fibula is positioned into the tibial defect and only the vessels of the contralateral fibula have to be anastomosed in microsurgical technique. For femoral defect reconstruction a bilateral free fibular graft can increase the primary stability and the increase of weight bearing can be accelerated. Osteosynthesis ensures primary stability during exercise. Considering the fact that most patients receive postoperative chemotherapy during treatment optimization studies (in the group of Ewing's sarcoma sometimes additional radiotherapy) an internal fixation (plating, Kirschner wires, etc.) is preferred to an external fixator. The aim is to reduce the risk of infection during times of pancytopenia [12]. Another reason for internal fixation is that generally a removal of the osteosynthesis is not required. Weight bearing is increased individually according to osseous integration of the fibular graft into the bone.

In our group of patients full weight-bearing of the affected limb was allowed after a median of 8 months corresponding to the results of Tomita et al. [28] and El-Gammal et al. [22, 23].

### 4.2. Postoperative Imaging

Plain radiographs in two planes are adequate for postoperative imaging. To the authors' opinion a general implementation of postoperative angiography or scintigraphy is not justified since it will not have surgical consequences in an asymptomatic patient (e.g., angiographically undetectable vascular anastomosis).

### 4.3. Limitations

A limitation of the bilateral fibular graft on one hand is the tumor location and on the other its expansion. Bilateral fibular grafts are favorable if the tumor is located meta- or diaphyseally in the long bones of the lower limb. Due to the rare occurrence of malignant bone tumors requiring diaphyseal resection the case number is low.

Tumor arthroplasty or allografts remain the reconstruction mode of choice if the epiphysis reveals tumor infiltration and neither a wide resection of the primary tumor nor a sufficient fixation of the interposition can be realized although there have been efforts even to implement biological reconstruction methods for osteoarticular defects [43].

Complications in the postoperative course after biological reconstruction cannot be avoided. They include in particular fractures of the fibular graft, non-union, and infection and are almost exclusively temporary [14, 24, 26, 41]. The causes of complications are versatile. Osseous integration of the graft during systemic chemotherapy is often delayed so that an increase in weight bearing must usually be performed over several months. In an asymptomatic patient this can lead to an unintended early increased weight bearing, which may result in a failure of fixation as seen in two of our patients.

In the group of Ewing's sarcomas often a neo- or adjuvant radiotherapy is indicated according to therapy optimisation studies and depending on the response rate to systemic chemotherapy. Those cases are predestined for a delayed union or pathological fractures, due to the well-known side effect of radiation therapy in musculoskeletal oncology [44] In our group of patients all three patients (100%) who had received radiotherapy suffered either a fracture of the fibular grafts (patient 5) or a fatigue fixation failure combined with a delayed union (patient 3 and 8). 

Infections are expected much less frequently in biological reconstructions than during the implantation of mega prosthesis. The cause of infection in biological reconstructions is either inadequate soft tissue coverage or osseous nonunion. Infection occurred in one of our patients who had received reconstruction of a proximal tibial defect (patient 7). Infection occurred despite a medial gastrocnemius flap and an additional fasciocutaneous flap and was accompanied by synchronous nonunion which was cured surgically.

Reported results in the literature are similar, while they are limited by low case numbers [23, 25, 26, 37, 41, 42]. There are only few reports on donor site morbidity [34]. Our patients did not suffer any. This corresponds to observations of Zaretski et al. [37].

## 5. Conclusions

In summary the biological reconstruction of metadiaphyseal defects of the long bones of the lower extremity with a bilateral fibular graft is a highly demanding surgical procedure aiming at limb salvage. Despite a high primary complication rate [1, 38, 42] resulting in secondary revisions, after managing complications and completion of osseous integration of the fibular grafts a permanent reconstruction can be assumed, making this procedure clearly superior to tumor arthroplasty. The authors therefore ask to consider biological reconstruction techniques as an adequate surgical option.

## Figures and Tables

**Table 1:** Patient characteristics: tumor resection and reconstruction with a bilateral fibular graft (*n* = 11).

Patient no.	Sex	Age at surgery	Diagnosis	Localisation	Tumor stage	Length of defect (cm)	Radiotherapy	Follow-up
1	w	43	chondrosarcoma	Femur	IIB	16	no	87
2	m	19	Ewing's sarcoma	Femur	IIB	16	no	144
3	w	15	Ewing's sarcoma	Femur	IIB	23	adjuvant	111
4	m	18	Ewing's sarcoma	Tibia	IIB	17	neoadjuvant	35
5	w	12	osteosarcoma	Tibia	IIB	11.5	no	120
6	m	13	Ewing's sarcoma	Femur	IIB	13.5	no	46
7	w	9	Ewing's sarcoma	Tibia	IIB	12	no	66
8	m	12	Ewing's sarcoma	Femur	IIB	16.5	adjuvant	63
9	m	14	Ewing's sarcoma	Tibia	IIB	24.5	no	36
10	w	4	Adamantinoma	Tibia	IIB	8	no	38
11	m	40	Adamantinoma	Tibia	IIB	8	no	12

**Table 2:** Results after tumor resection and reconstruction with a bilateral fibular graft (*n* = 11).

Patient no.	Resection	Regression grade^a^	Complications	Time until full weight (months)	Outcome	MSTS (1993)
1	R0	*n*	None	8	NED	70%
2	R0	2	Bleeding from anastomosis	5	NED	100%
3	R0	3	Plate failure and delayed union	9	NED	87%
4	R0	1	Fibular graft fracture, conservative treatment	18	DOD	60%
5	R0	3	Infection, and nonunion	8	NED	87%
6	R0	1	None	7	DOD	93%
7	R0	1	None	9	NED	93%
8	R0	4	Plate failure and delayed union	9	NED	80%
9	R0	3	None	13	NED	67%
10	R0	*n*	None	4	NED	87%
11	R1	*n*	None	7	NED	100%

^a^Referred to Salzer-Kuntschik [32]. *n*: not applicable, NED: no evidence of disease, DOD: dead of disease.
